# Molecular Characterization of Human Papillomavirus Type 159 (HPV159)

**DOI:** 10.3390/v13081668

**Published:** 2021-08-23

**Authors:** Iva Marković, Lea Hošnjak, Katja Seme, Mario Poljak

**Affiliations:** 1 Division of Molecular Biology, Ruđer Bošković Institute, Bijenička Cesta 54, 10000 Zagreb, Croatia; imarkov@irb.hr; 2 Institute of Microbiology and Immunology, Faculty of Medicine, University of Ljubljana, Zaloška Cesta 4, 1105 Ljubljana, Slovenia; lea.hosnjak@mf.uni-lj.si (L.H.); katja.seme@mf.uni-lj.si (K.S.)

**Keywords:** human papillomavirus, *Betapapillomavirus*, phylogenetic characterization, viral load, prevalence, tissue tropism

## Abstract

Human papillomavirus type 159 (HPV159) was identified in an anal swab sample and preliminarily genetically characterized by our group in 2012. Here we present a detailed molecular in silico analysis that showed that the HPV159 viral genome is 7443 bp in length and divided into five early and two late genes, with conserved functional domains and motifs, and a non-coding long control region (LCR) with significant regulatory sequences that allow the virus to complete its life cycle and infect novel host cells. HPV159, clustering into the cutaneotropic *Betapapillomavirus* (*Beta*-PV) genus, is phylogenetically most similar to HPV9, forming an individual phylogenetic group in the viral species *Beta*-2. After testing a large representative collection of clinical samples with HPV159 type-specific RT-PCR, in addition to the anal canal from which the first HPV159 isolate was obtained, HPV159 was further detected in other muco-cutaneous (4/181, 2.2%), mucosal (22/764, 2.9%), and cutaneous (14/554, 2.5%) clinical samples, suggesting its extensive tissue tropism. However, because very low HPV159 viral loads were estimated in the majority of positive samples, it seemed that HPV159 mainly caused clinically insignificant infections of the skin and mucosa. Using newly developed, highly sensitive HPV159-specific nested PCRs, two additional HPV159 LCR viral variants were identified. Nevertheless, all HPV159 mutations were demonstrated outside important functional domains of the LCR, suggesting that the HPV159 viral variants were most probably not pathogenically different. This complete molecular characterization of HPV159 enhances our knowledge of the genome characteristics, tissue tropism, and phylogenetic diversity of *Beta*-PVs that infect humans.

## 1. Introduction

Papillomaviruses (PVs) are a diverse group of heterogeneous small DNA viruses that infect a wide range of vertebrate species and are associated with the development of various neoplastic changes in the cutaneous and mucosal epithelia [1,2,3]. PVs’ genetic material consists of circular double-stranded DNA of approximately 8000 base pairs, divided into three distinct regions: two coding and one non-coding [4,5,6].

Based on their L1 genomic sequences, human PVs (HPVs) are classified into five genera (*Alphapapillomavirus* (*Alpha*-PV), *Betapapillomavirus* (*Beta*-PV), *Gammapapillomavirus* (*Gamma*-PV), *Mupapillomavirus* (*Mu*-PV), and *Nupapillomavirus* (*Nu*-PV)) [5,7].

The PVs’ coding region, which represents the majority of the viral genome, is divided into E (early) and L (late) genes, according to the spatiotemporal patterns of expression over the viral life cycle [6,8]. Most HPVs have six different early genes: E1, E2, E4, E5, E6, and E7, which code for the proteins that are responsible for viral propagation (E1 and E2 for replication and transcription, respectively), binding to cytokeratins (E4), cellular transformation (E5-binding to receptors of growth factors), and oncogenesis (E6 and E7) [1,6,9,10,11]. Initial studies indicated that *Beta*-PVs lack the E5 gene in the early viral region. However, recent studies showed that this gene can also be found in the form of the E8^E2C transcript, with very similar functions as the E5 protein of mucosal *Alpha*-PV high-risk HPV types [6]. PVs’ late viral region contains two genes: L1 (large capsid protein) and L2 (small capsid protein), forming 80 to 90% and 10 to 20% of the complete protein capsid, respectively. The non-coding long control region (LCR) is located between the L1 and E6 genes and contains most of the regulatory elements that are included in the viral processes of DNA replication and transcription. In comparison to mucosal *Alpha*-PV high-risk HPV types, *Beta*-PVs have a shorter LCR, resulting in shorter HPV genomes [6].

Unlike *Alpha*-PVs, *Beta*- and *Gamma*-PVs can persist on the skin for a longer time and they generally cause clinically insignificant viral infections [3,12,13]. These infections, especially with *Beta*-PVs, are common in early childhood and can be transmitted to parents because family members are often infected with the same PV types [6,13,14]. In addition, the *Beta*-PV genus also includes HPVs that are associated with the onset of benign and malignant cutaneous neoplasms in immunocompromised individuals and patients with the rare hereditary disease epidermodysplasia verruciformis (EV). Specifically, in comparison to healthy individuals, immunocompromised individuals, including organ/tissue transplant recipients, are more likely to be infected with *Beta*-PV types and have an increased risk of developing non-melanoma skin cancer [6,15,16]. Moreover, in human immunodeficiency virus (HIV)–positive patients, these viruses can frequently be found in their hair follicles, suggesting that the bulge area of the outer layer of the hair follicle is one of the main reservoirs of *Beta*-PVs [6].

In contrast to *Alpha*-PV genera in which the viral variants of the same HPV type (e.g., HPV16) differ in their pathogenic potential, to the best of our knowledge, no such connections have previously been described for other HPV genera. Moreover, so far, the existence of *Beta*-PV viral variants has been described for only a few HPV types (HPV5, 17, 38, 104, and 120) [7].

The aims of this study were to perform a complete genomic characterization and phylogenetic evaluation of the novel *Beta*-PV type, HPV159, which was identified and preliminarily genetically characterized by our group in 2012 [17]. In addition, a large representative collection of clinical samples was tested using in-house HPV159 type-specific RT-PCR to establish HPV159’s tissue tropism, genetic diversity, and its potential clinical relevance.

## 2. Materials and Methods

### 2.1. HPV159 Reference Isolate

The HPV159 reference isolate was obtained from an anal swab sample from a 36-year-old immunocompetent man from Slovenia that participated in a study on the prevalence of HPV types in the anal canal of individuals with a history of same-sex intercourse. The complete viral genome was amplified from a rolling circle amplification (RCA) product of the original anal swab sample DNA isolate using two overlapping PCR fragments (7348 bp and 761 bp), as described previously [17]. Two reference clones, covering the full genome of HPV159, were deposited in July 2012 in the International HPV Reference Center, Heidelberg, Germany, where their sequences were independently confirmed, and the novel type was officially named HPV159 in September 2012.

### 2.2. Sample Collection

This study included DNA isolates from a representative collection of 1499 clinical samples obtained from the same number of individuals with a clinically normal oral cavity (*n* = 401), oral and oropharyngeal squamous cell carcinoma (SCC; *n* = 135), a clinically normal nasopharynx (*n* = 60), a clinically normal larynx (*n* = 10), laryngeal verrucous SCC (*n* = 10), a cytologically normal cervix (*n* = 60), cervical stratified mucin-producing intraepithelial lesions (SMILE; *n* = 37), penile warts (*n* = 51), a clinically normal anal canal and anal edge (*n* = 121), perianal and anal warts (*n* = 60), eyebrow hair follicles (*n* = 367), common warts (*n* = 86), cutaneous SCC (*n* = 50), and basal cell carcinoma (BCC; *n* = 51).

#### Ethics Statement

The retrospective clinical samples included in this study (*n* = 1130) were collected during our previous studies, which were done in compliance with the Helsinki Declaration and regulations of the Ethics Committee of the Slovenian Ministry of Health (consent references 34/11/06, 83/11/09, 174/05/09, 97/11/09, and 100/12/09). Written informed consent was also obtained from each patient. The use of archival tissue samples (*n* = 369), which were retrieved from the collection of formalin-fixed paraffin-embedded (FFPE) tissue samples of the Institute of Pathology, Faculty of Medicine, University of Ljubljana, was approved by the Institutional Review Board of the Institute of Pathology and the Institute of Microbiology and Immunology, Faculty of Medicine, University of Ljubljana, prior to the start of the study. In accordance with Slovenian legislation, no informed consent is needed for research on archival samples. To protect the patients’ identities, all samples used in the study were coded and tested anonymously. The only available data were each patient’s sex, age, and immune status (if collected during the original study).

### 2.3. Molecular Determination and Phylogenetic Analysis of HPV159

For the in silico molecular analysis of the HPV159 viral genome and identification of genes encoding viral proteins, the Vector NTI Advance v11.5.4 program (Invitrogen, Carlsbad, CA, USA) and the online ORF finder tool (http://www.ncbi.nlm.nih.gov/gorf/gorf.html, accessed on 6 July 2020), which allows for the identification of viral open reading frames (ORFs) with the Protein BLAST algorithm (https://blast.ncbi.nlm.nih.gov/Blast.cgi, accessed on 6 July 2020) of the UniProtKB/Swiss-Prot database (https://www.uniprot.org/uniprot/?query=reviewed:yes, accessed on 6 July 2020), were used, as described previously [17]. In compliance with the agreement of the HPV community, the circular viral genome was cut at the start of the E6 ORF. In addition, individual regulatory sequences and the most conserved functional domains within viral proteins were identified through several available online applications, such as GPMiner (http://gpminer.mbc.nctu.edu.tw/index.php, accessed on 6 July 2020), SIGSCAN software v4.05 [18], and Patch 1.0 (http://gene-regulation.com/cgi-bin/pub/programs/patch/bin/patch.cgi, accessed on 6 July 2020). The putative polyadenylation sites for early and late viral mRNAs were determined with the web application Poly (A) Signal Miner (http://dnafsminer.bic.nus.edu.sg/PolyA.html, accessed on 6 July 2020) [19]. Finally, the presence of leucine zippers in the DNA-binding domain of the E2 protein was verified using the online application 2ZIP-Server (http://2zip.molgen.mpg.de/index.html, accessed on 6 July 2020) [20].

The phylogenetic analysis was based on the complete L1 gene sequence of HPV159 and corresponding nucleotide sequences of already classified and officially recognized HPV types from the *Alpha*- (*n* = 64), *Beta*- (*n* = 47), *Gamma*- (*n* = 78), *Mu*- (*n* = 3), and *Nu*-PV (*n* = 1) genera. Nucleotide sequences of the aforementioned L1 genes were obtained from the database of reference PV types, namely, Papillomavirus Episteme (PaVE, https://pave.niaid.nih.gov/, accessed on 7 July 2021 [7]), and aligned using the MUSCLE algorithm, which is part of the MEGA7.0.26 package [21]. The same software package [21] and the maximum likelihood algorithm (Institute for Genomics and Evolutionary Medicine, Temple University, Philadelphia, PA, USA) were also used to create the phylogenetic tree, which was finally presented using the FigTree program 1.3 (http://tree.bio.ed.ac.uk/software/figtree/, accessed on 7 July 2021).

For further comparison of the reference HPV159 isolate with the phylogenetically closest HPV type (HPV9), similarities between the nucleotide/amino acid sequences of individual genes/proteins were compared and calculated using the BioEdit Sequence Alignment Editor v7.2.6.1 (Ibis Therapeutics, Carlsbad, CA, USA) and the online EMBOSS Water Pairwise Sequence Alignment tool (http://www.ebi.ac.uk/Tools/psa/emboss_water/, accessed on 8 July 2021).

### 2.4. Prevalence and Potential Clinical Significance of HPV159

For amplification of the 112 bp fragment of the HPV159 L1 gene, primers (HPV159-RT-FW-New: 5′-CGAGTTCAAAGCACGGATG-3′, HPV159-RT-RW-New: 5′-CATCCTGAGAGCGAACATCA-3′) and a probe (HPV159-RT-P-New: 5′-GTGATCGTTTGCTGACAGTAGGAC-3′) for real-time PCR (RT-PCR) were designed using the web-based application Primer3 v0.4.0 (http://bioinfo.ut.ee/primer3-0.4.0/, accessed on 13 April 2019). The chemical/thermodynamic properties and specificity of the proposed primers were additionally evaluated by the online applications Net Primer (http://www.premierbiosoft.com/netprimer/, accessed on 13 April 2019) and BLAST (NCBI, U.S. National Library of Medicine, Bethesda, MD, USA), respectively.

To perform the HPV159 RT-PCR, the aforementioned primers were used in combination with the commercially available kit LightCycler480 Probes Master (Roche Diagnostics, Mannheim, Germany). Each reaction mixture (25 μL) contained 10 μL of LC480 Probes Master (2×; Roche Diagnostics), 0.2 μL of each primer (50 μM) and probe (20 μM; FAM), 4.4 μL of sterile nuclease-free water (Qiagen, Hilden, Germany), and 5 μL of sample DNA (optimal concentration of added DNA = 4 ng/μL). The cycling conditions on the LightCycler 480 Instrument II (Roche Diagnostics) were as follows: 95 °C for 10 min, followed by 40 amplification cycles of 95 °C for 10 s, 60 °C for 30 s, and 72 °C for 1 s (data gain on the 530 nm channel). A final step consisted of cooling the reaction mixture to 40 °C with a 30 s hold.

The analytical sensitivity of the test (at least 10 viral copies/reaction) was determined using the plasmid dilutions that contained the HPV159 genome in concentrations of 1 to 10^9^ viral DNA copies/reaction in the presence of 100 ng of human DNA. The correlation coefficient (R^2^) and amplification efficiency (E) were estimated at 0.9992 and 96.7%, respectively.

The quality and concentration of the extracted DNA were determined using a quantitative RT-PCR amplification of a 150 bp fragment of the human beta-globin gene using beta-403f/beta-532r primers, as described previously [7]. In line with the published work of Hazard et al. (2006) [22], we hypothesized that one human cell possesses 6.6 pg of genomic DNA. Consequently, the HPV159 viral loads were expressed as the ratio between viral genome copy numbers and human cell count.

### 2.5. Determination of HPV159 Long Control Region Viral Variants

The HPV159 viral variants were determined based on the most diverse part of the HPV genome, the LCR region, using the newly developed semi-nested PCR primers (HPV159-LCR-FW (5′-GAGCCTACAGAACGTGAAG-3′), HPV159-LCR-RV (5′-AGGAATAGTCAAGGTATCTGC-3′), HPV159-LCR-FW-N1 (5′-GATAGCGGTGCTCAATAAA-3′), and HPV159-LCR-RV-N1 (5′-AGGAATAGTCAAGGTATCTGC-3′)), which were used in the following combination: HPV159-LCR-FW/HPV159-LCR-RV (outer PCR; length of the PCR product: 666 bp), HPV159-LCR-FW/HPV159-LCR-RV-N1 (semi-nested PCR1; 449 bp), and HPV159-LCR-FW-N1/HPV159-LCR-RV (semi-nested PCR2; 449 bp).

The described HPV159-nested PCRs were based on the commercially available FastStart High Fidelity PCR System (Roche Diagnostics) and Veriti Thermal Cycler (Applied Biosystems, Foster City, USA). The reaction mixture (25 μL) contained 0.5 μL of the dNTP mix (10 mM; Roche Diagnostics), 2.5 μL of 10× FastStart High Fidelity Reaction Buffer (+1.8 mM MgCl_2_; Roche Diagnostics), 0.2 μL of each primer (50 μM), 0.25 μL of FastStart High Fidelity Enzyme Blend (5 U/μL; Roche Diagnostics), 16.35 μL of sterile nuclease-free water (Qiagen), and 5 μL of sample DNA (optimal concentration of added DNA = 4 ng/μL). The cycling conditions were as follows: 95 °C for 2 min, followed by 35 amplification cycles of 95 °C for 30 s, 50 °C for 30 s, and 72 °C for 1 min. A final step consisted of incubation at 72 °C with a 7 s hold, and final cooling of the reaction mixture to 4 °C.

The obtained PCR products were gel purified and further processed for Sanger sequencing, as described previously [23,24]. Purification of sequencing products was performed according to the instructions of the commercially available BigDye XTerminator Purification Kit (Applied Biosystems) set of chemicals. Subsequently, the reaction plates were placed in an ABI3500 Genetic Analyzer (Applied BioSystems) computer system, which allowed for automatic assessment of nucleotide sequences and was run with the BDx_FastReadSeqPOP7 protocol. The nucleotide sequences obtained were assembled using the aforementioned Vector NTI Advance v11.5.4 (Invitrogen) program and compared with the HPV159 reference genome sequence (GenBank acc. no. HE963025).

In samples with low viral DNA concentrations, RCA, using the Ilustra TempliPhiTM 100 Amplification Kit (GE Healthcare, Amersham, UK) and Veriti 96-Well Thermal Cycler (Applied Biosystems), was performed prior to determination of the HPV159 LCR viral variants in order to enrich the circular DNA genomes [25].

## 3. Results and Discussion

The cloned complete HPV159 viral genome (HE963025), which was originally amplified from an anal swab sample from a man with a history of same-sex intercourse, has been stored at the Reference Center for Papillomaviruses in Stockholm, Sweden, since July 2012, and the preliminary results of its genetic characterization were published in 2013 [17]. This article reports the complete molecular analysis of the HPV159 genome, its phylogenetic placement within the PV family, tissue tropism, potential clinical relevance, and genetic diversity.

**Figure 1:**
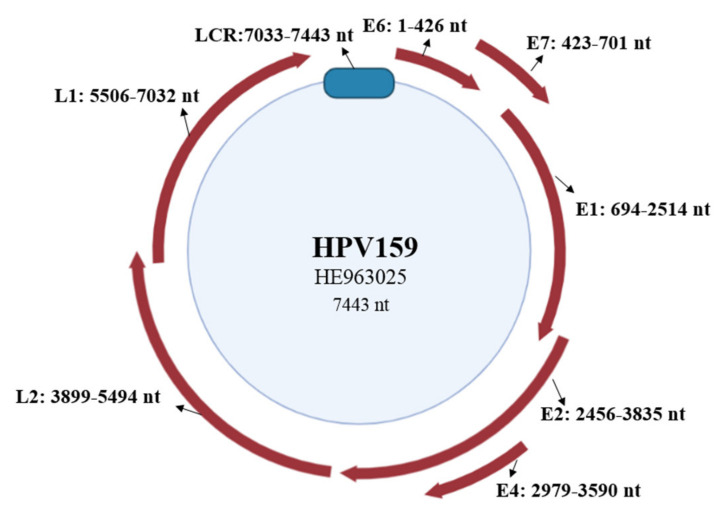
HPV159 genome organization. The positions of viral genes (E6, E7, E1, E4, E2, L2, and L1) and the non-coding long-control region (LCR) are marked with red arrows and a blue rounded rectangle, respectively.

The HPV159’s viral genome is 7443 bp in length and thus corresponds to other HPV types, whose genetic material consists of double-stranded circular DNA of approximately 8000 bp [17]. Unlike *Alpha*-PV genomes, which usually contain eight viral genes [3,4,5] within early and late coding regions, the HPV159 genome consists of seven ORFs (E1, E2, E4, E6, E7, L1, and L2), as is characteristic for *Beta*-PVs (Figure 1) [26]. Nevertheless, the HPV159 genome lacks the conventional E5 ORF, and it contains an alternative form of the aforementioned gene, the E8^E2C transcript [6].

As shown in Figure 1, the HPV159 LCR was located between the L1 and E6 ORFs and consisted of 410 bp (nt 7033–7443). The LCR included characteristic DNA sequences that are necessary for the regulation of viral transcription and replication [27]. The 5′ end of the HPV159 LCR thus contained a putative polyadenylation site (AATAAA, nt 7048–7053), which is necessary for processing late viral transcripts and adding a polyadenylation tail to their 3′ ends [27], while the 3′ end of the HPV159 LCR was the binding site for the E1 protein (TTGTGGTTAACAACAATCAT, nt 7341–7360), which most likely represents the origin of replication, and regulatory transcription factors, such as AP-1, NF-1, Sp1, TFIID, and C/EBP [28]. The HPV159 LCR sequence additionally had three consensus palindromic E2-binding sites (ACCGATAGCGGT, nt 7032–7043; ACCGCGCCCGTT, nt 7133–7144; and ACCGATAACGGT, nt 7289–7300) that are important for transcription activation and repression, initiation of replication, and viral genome maintenance [29]. Moreover, this sequence also included two putative TATA motifs (TATAAA, nt 7154–7159; nt 7405–7410) of the early promoter of viral gene transcription.

The HPV159 E1 protein was the largest HPV159 protein (606 amino acids; aa), which is consistent with the average size of other PV E1 proteins, varying from 600 to 650 aa. The 5′ end of the HPV159 E1 protein contained a conserved ATP-binding site (GPPDTGKS, aa 434–441) that makes possible its role as the ATP-dependent hexamer DNA helicase. In all PV genomes, this viral structure is an essential part for replication and amplification of viral episomes in the nuclei of infected keratinocyte cells [28,30], which, in HPV159, binds to a long, incomplete 18 bp palindromic region within the LCR ori site (TTGTGGTTAACAACAATCAT, nt 7341–7360). The 3′ end of the E1 protein is the least conserved PV protein segment and contains several short amino acid motifs [28]. In HPV159, these sequences included a nuclear localization signal (NLS), consisting of a highly conserved KRK sequence located at 80–82 aa, followed by the leucine-rich core signal (LRSVLAALFW) at amino acid positions 267–277. This NLS also contained a binding site (RRL, located at 109–111 aa) that allows the cyclin E/cyclin A to bind within a cyclin-dependent kinase complex, cdk2, resulting in phosphorylation of this site and binding of nuclear transport carrier receptors (importins and exportins) [5,28,31,32,33,34,35].

Consistent with the conventional lengths of PV E2 proteins (350–500 aa), HPV159 E2 contained 459 aa and had two functional domains that allowed for the regulation of early viral gene expression and a DNA binding protein, which was bound to specific consensus motifs that were located in the LCR region of the viral genome (ACCGATAGCGGT, 7032–7043 nt; ACCGCGCCCGTT, nt 7133–7144; and ACCGATAACGGT, nt 7289–7300). The conserved 5′ end of the HPV159 E2 region contained a leucine zipper motif (Lx_6_Lx_6_Lx_7_L, LSDRFNALQETLMELYEAGREDL, 4–26 aa) that slightly differed from the conventional PV motif, which usually appears as Lx_6_Lx_6_Lx_6_L [8,30,33,34]. In addition, the HPV159 E2 protein, as in most *Beta*-PVs, had a highly conserved RSQSRSQSRSRSRSRS (318–333 aa) motif, which is phosphorylated by protein kinase A (PKA) activity [33].

The HPV159 E4 protein is fully encoded within the E2 ORF and contains 203 aa, as well as a start codon (ATG) between 2988 and 2990 bp [8,17,31]. At the 3′ end, the HPV159 E4 protein contained a leucine-cluster domain LLSLVLRHLL (22–31 aa), which is important for keratin binding, and the 5′ end allows the multimerization of the protein [8,30,36]. Unlike other viral proteins, HPV159 E4 contained a significantly higher amount of proline (19.7%).

The HPV159 E6 protein consisted of 141 aa and contains two preserved zinc-finger domains, identified based on the highly conserved consensus sequences CxxC(x)_30_CxxC and CxxC(x)_29_CxxC, at positions 27–64 aa and 101–137 aa, separated by 36 aa, respectively, that in PVs bind zinc ions and are responsible for intracellular stability and conformation [6]. In addition, the E3 ubiquitin ligase, also known as E6AP, which is the first protein to interact with PVs’ E6 [6,37,38], was recognized in HPV159 based on the consensus motif of LIDLL at 20–24 aa.

The HPV159 E7 protein was composed of 92 aa. In PVs, E7 is a helper protein, which is primarily found in the nuclei of infected cells, where it reprograms the cellular environment to be suitable for viral replication [6,38,39]. It also had an LxCxE motif (in HPV159: LHCYE, 24–48 aa) for binding/interacting with the tumor suppressor retinoblastoma protein (pRB) and allows for stable binding of other proteins from the same family, such as p107 and p130 [5,6]. The 5′ end of the HPV159 E7 protein additionally contained a slightly modified zinc-finger domain (CxC(x)_29_CxxC, aa 50–85), which in PVs enhances the formation of viral protein dimers and allows proper functioning of their virions [5,19,31,39].

PVs’ late viral proteins, L1 and L2, are required for genome packaging and viral cell formation [40,41,42,43]. As mentioned above, the L1 gene encodes for the large capsid protein unit and has the most conserved ORF of all viral proteins [40]. The capsid protein L2 plays an essential role in PVs’ assembly and infectious processes [41,42,43]. The HPV159 L1 and L2 proteins contain 508 and 531 aa, respectively, whereas the 5′ region of the L2 protein is characterized by a PxxP residue representing the L1-binding region [43,44,45], located at 497–500 aa with the PEAP motif, the 3′ end of the same protein contains a furin-cleavage motif with a consensus sequence RxK/RR (RTKR, 6–9 aa). Moreover, several GxxxG motifs, whose mutations are important for endosomal retention, were characterized at 13–17, 355–359, and 383–387 aa of the HPV159 L2 protein, respectively.

In summary, the results of the molecular in silico analysis of the HPV159 reference genome suggested that the viral genome encodes significant viral proteins with conserved functional domains and motifs and the non-coding LCR region with important regulatory DNA sequences that enable the virus to complete its life cycle and infect novel host cells.

**Figure 2:**
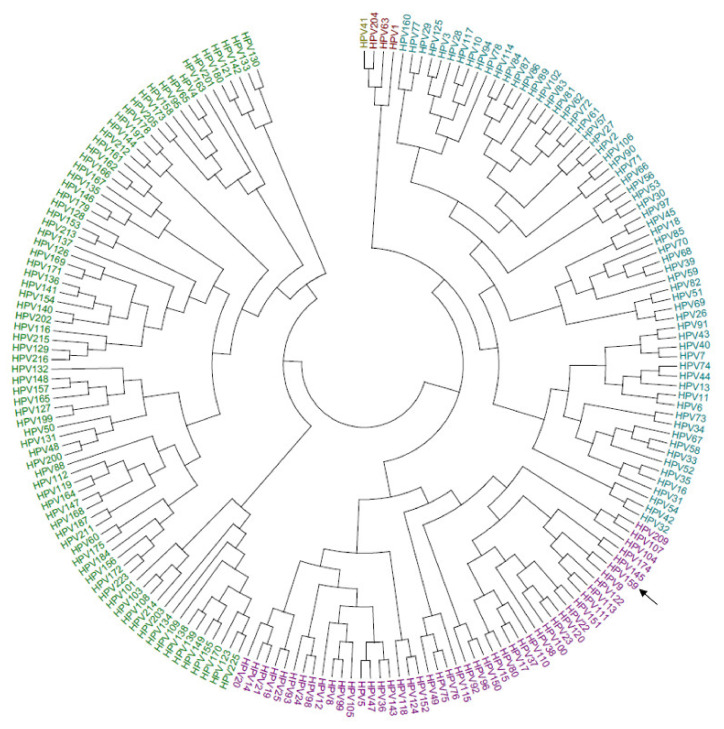
A phylogenetic tree that was obtained from the available nucleotide sequences of the L1 gene of all HPV types (https://pave.niaid.nih.gov/, accessed on 7 July 2021). The black arrow marks HPV159. *Alpha*-PVs are marked with blue, *Beta*-PVs with purple, *Gamma*-PVs with green, *Mu*-PVs with dark red, and *Nu*-PVs with yellow.

Based on the phylogenetic tree of L1 nucleotide sequences of all officially characterized HPV types as of July 2021 (Figure 2), HPV159 belongs to the cutaneotrophic genus *Beta*-PV, species *Beta*-2, confirming previously published results [17]. As shown in Figure 2, HPV159 is most similar to HPV9. Therefore, their similarity was further investigated using pairwise nucleotide and amino acid sequence comparisons of individual ORFs, as shown in Table 1. The greatest similarity between the two HPV types was shown in the nucleotide sequences of the E7 ORF (81.6%) and the amino acid sequences of the L1 protein (94.5%). Taken together and according to the literature data [46], the high similarity of nucleotide/amino acid sequences of these two HPV types could suggest their evolutionary divergence from a common ancestor.

Human *Beta*-PVs are typically associated with cutaneous infections, mainly in immunocompromised individuals and patients suffering from the hereditary disease EV, and they rarely cause visible skin lesions [8]. This study investigated the presence of the novel HPV159 in a representative collection of 1499 clinical specimens of apparently healthy skin and mucosa, as well as various HPV-related benign and malignant neoplasms, using the newly developed highly sensitive and specific HPV159 type-specific RT-PCR. As shown in Table 2, a total of 40/1499 (2.7%) clinical samples were determined to be HPV159-positive, with HPV159 being detected in a sample of perianal/anal warts (1/60, 1.7%), as well as in various swab samples of clinically normal mucosa and mucocutaneous tissues, including the oral cavity (20/401, 4.9%), larynx (2/10, 20.0%), and anal canal and anal edge (3/121, 2.5%), similarly to some previous studies [47,48,49,50]. The presence of *Beta*-PVs was also demonstrated in eyebrow hair follicles of healthy adults [6,51], leading to the hypothesis that the bulge area of the outer layer of a hair follicle is one of the main *Beta*-PV reservoirs. In accordance with previously reported studies, in our collection of eyebrow hair follicle samples, 14/367 (3.8%) were positive for HPV159. Nevertheless, low HPV159 viral loads (0.001532 to 0.95568 viral copies/10^4^ cell; Table 3) were estimated in the oral cavity and hair follicles, suggesting that HPV159 mostly causes clinically insignificant cutaneous and mucosal infections [49,52,53,54].

In order to evaluate the HPV159 LCR viral variants and their potential association(s) with the particular lesion type(s), a total of 40 HPV159-positive samples were further tested using an in-house semi-nested LCR PCR. Because most of the HPV159-positive samples were FFPE archival tissue samples or cutaneous/mucosal swabs with fragmented DNA and/or low viral loads, the analysis focused on the non-coding LCR region and utilized newly designed highly sensitive (at least 10 viral copies/reaction) nested PCRs. Although we adjusted and optimized the PCR performance characteristics for the clinical sample type, the sequence of complete HPV159 LCR region was obtained from three HPV159-positive samples only. As shown in Table 4, the HPV159 isolate obtained from the anal swab sample (A395Re; GenBank acc. no. MZ147848) was identical to the HPV159 reference isolate (HE963025), whereas the other two samples that were taken from eyebrow hair follicles (1-o-1, MZ147849; 17-o-723, MZ147850) differed from the HPV159 reference sequence in six (6/410, 1.46%) and eight (8/410, 1.95%) nucleotide sites, respectively. All HPV159 mutations were detected beyond important functional HPV159 LCR domains, suggesting that the HPV159 viral variants were most probably not pathogenically different, as was previously described for some *Alpha*-PVs. However, due to the low number of samples included in the analysis of viral variants, our results warrant further research.

## 4. Conclusions

The molecular in silico analysis showed that the viral genome of the *Beta*-PV HPV159 encoded for seven ORFs (E1, E2, E4, E6, E7, L1, and L2) and a highly conserved non-coding LCR region, which is necessary for regulation of viral transcription and replication. After testing a large representative collection of clinical samples with HPV159 type-specific RT-PCR, in addition to the anal canal from which the first HPV159 isolate was obtained, our study further detected HPV159 in other muco-cutaneous, as well as in mucosal and cutaneous clinical samples, suggesting its broad tissue tropism. Because HPV159 viral loads were low in all positive samples, it seemed that HPV159 mostly caused clinically insignificant infections. All HPV159 mutations were demonstrated outside important functional compartments of the LCR, suggesting that the HPV159 viral variants were most probably not pathogenically different. The complete molecular characterization of HPV159 enhances our knowledge of the genome characteristics, tissue tropism, and phylogenetic diversity of *Beta*-PVs that infect humans.

## Figures and Tables

**Table 1:** Percentage similarity between individual viral genes of HPV159 and the phylogenetically most related HPV type: HPV9.

HPV159 ORFs		HPV9 ORFs’ Sequence Similarity (%)
**E6**	nt	73.3
	aa	83.0
**E7**	nt	81.6
	aa	89.1
**E1**	nt	80.5
	aa	90.7
**E2**	nt	77.6
	aa	84.6
**E4**	nt	73.2
	aa	72.9
**L1**	nt	79.6
	aa	94.5
**L2**	nt	75.7
	aa	92.9

Abbreviations/legend: nt = nucleotide, aa = amino acid.

**Table 2:** Determination of HPV159 infection in a collection of mucosal, muco-cutaneous, and cutaneous clinical specimens.

Tissue Type	Sample Type	No. of Samples Tested	No. of Positive Samples	Prevalence (%)
**Mucosal**	Oral cavity: normal tissue (swabs)	401	20	4.9
	Oral cavity and oropharynx: SCC (FFPE)	135	0	0
	Nasopharynx: normal tissue (swabs)	60	0	0
	Larynx: normal tissue (swabs)	10	2	20
	Larynx: verrucous SCC (FFPE)	10	0	0
	Cervix: normal cytology (swabs)	60	0	0
	Cervix: SMILE (FFPE)	37	0	0
	Penis: genital warts (fresh tissue)	51	0	0
**Muco-cutaneous**	Anal canal and anal edge: normal tissue (swabs)	121	3	2.5
	Perianal skin, anus: genital warts (fresh tissue)	60	1	1.7
**Cutaneous**	Eyebrows (hair follicles)	367	14	3.8
	Skin: common warts (FFPE)	86	0	0
	Skin: SCC (FFPE)	50	0	0
	Skin: BCC (FFPE)	51	0	0
**Total**		1499	40	2.7

Abbreviations/legend: FFPE = formalin-fixed, paraffin-embedded tissue; SCC = squamous cell carcinoma; BCC = basal cell carcinoma; SMILE = stratified mucin-producing intraepithelial lesions.

**Table 3:** Viral load in HPV159-positive eyebrow hair follicles and the oral cavity.

Tissue Type	Sample Type	Sample ID	Viral Load (Viral Copies/10^4^ Cells)
Cutaneous	Eyebrows (hair follicles)	626	0.002
		625	0.002
		622	0.003
		596	0.01
		594	0.003
		303	0.003
		254	0.03
Mucosal	Clinically normal oral cavity (swabs)	249	0.02
		73	0.01
		72	0.04
		105	0.09
		75	0.04
		87	0.03
		71	0.06
		85	0.30
		37	0.05
		89	0.46
		86	0.16
		82	0.96
		63	0.88

**Table 4:** HPV159 long control region viral variants identified in three HPV159-positive clinical samples (two eyebrow hair follicles and one anal swab). The nucleotide sites of the HPV159 reference isolate (GenBank acc. no. HE963025) at which nucleotide alterations occurred in the clinical samples are marked at the top of the table. Genomic sites where no changes were observed are marked with a dot.

		Clinical Samples		
Nucleotide Sites in LCR	HPV159 (HE963025)	A395Re (MZ147848; Anal Canal)	1-o-1 (MZ147849; Eyebrows)	17-o-723 (MZ147850; Eyebrows)
7184	T	.	.	A
7234	C	.	.	A
7284	G	.	.	A
7288	G	.	.	A
7316	G	.	C	.
7336	A	.	G	.
7338	T	.	G	.
7339	A	.	G	.
7341	T	.	C	.
7365	C	.	.	T
7429	A	.	.	G
7431	T	.	.	A
7437	C	.	.	A
7439	G	.	A	.

## Data Availability

The authors confirm that the data supporting the findings of this study are openly available in the GenBank database at https://www.ncbi.nlm.nih.gov/genbank/, accessed on 29 June 2021, under accession numbers MZ147848 (HPV159 LCR, isolate A395Re), MZ147849 (HPV159 LCR, isolate 1-o-1), and MZ147850 (17-o-723, isolate 17-o-723).
